# Stability, Continuity, and Bi-Directional Associations of Parental Feeding Practices and Standardized Child Body Mass Index in Children from 2 to 12 Years of Age

**DOI:** 10.3390/nu11081751

**Published:** 2019-07-30

**Authors:** Janina Eichler, Ricarda Schmidt, Tanja Poulain, Andreas Hiemisch, Wieland Kiess, Anja Hilbert

**Affiliations:** 1 Leipzig University Medical Center, Integrated Research and Treatment Center AdiposityDiseases, Medical Psychology and Medical Sociology, Psychosomatic Medicine and Psychotherapy, Philipp-Rosenthal-Strasse 27, 04103 Leipzig, Germany; 2 LIFE Child Leipzig Research Center for Civilization Diseases, Leipzig University, Philipp-Rosenthal-Strasse 27, 04103 Leipzig, Germany; 3 Department of Women and Child Health, Hospital for Children and Adolescents and Centre for Paediatric Research (CPL), Leipzig University, Liebigstrasse 20a, 04103 Leipzig, Germany

**Keywords:** feeding practices, body mass index, child weight, children, stability, bi-directional

## Abstract

(1) Background: Parental feeding practices are related to child body mass index (BMI, kg/m^2^) and seem to be a consequence rather than cause of child BMI, but research so far is limited. Stability and continuity of feeding practices, probably explaining changes in food intake and child BMI, remain to be poorly examined. (2) Methods: Feeding practices (i.e., restriction, food as reward, pressure to eat, monitoring) assessed via the Child Feeding Questionnaire, child age, standardized BMI (*z*BMI), and socio-economic status were measured annually at multiple visits (range 2–8) in a population-based longitudinal cohort study of 1512 parents with their children aged 2 to 12 years. Stability, continuity, and bi-directionality of feeding practices and child *z*BMI were calculated using correlation coefficients, paired *t* tests, and cross-lagged panels, respectively. (3) Results: Feeding practices and child *z*BMI showed moderate to high stability. While continuity was high for restriction, minor temporal changes were observed for other feeding practices and child *z*BMI. Cross-lags indicated that child *z*BMI predicted restriction, pressure to eat, and monitoring, while food-rewarding predicted child *z*BMI only minorly. (4) Conclusions: Parents seem to adapt feeding practices to child *z*BMI with the exception of food-rewarding.

## 1. Introduction

Overweight and obesity in childhood and adolescence are an increasing public health concern [1,2,3]. While in 1980 16.9% of boys and 16.2% of girls were overweight or obese, international prevalence rates rose to 23.8% and 22.6% in 2013, respectively [4]. Overweight and obesity in childhood are predictive of overweight and obesity in adulthood and the onset of related physical (e.g., cardiovascular syndrome, metabolic syndrome) and psychological comorbidities (e.g., depressive disorders, attention-deficit hyperactivity disorder; [5,6,7]). Fundamentally, overweight and obesity are caused by an imbalance between energy intake and expenditure [8], but their etiologies are complex [1,9]. Notably, the increasing prevalence of overweight and obesity suggests that environmental and genetic factors are involved in the development of childhood overweight and obesity. A change in these environmental factors could promote a reduction in the obesity epidemic [10]. One of the potential environmental factors influencing energy intake in children are parental feeding practices [11], which include parental beliefs, attitudes, and practices concerning child feeding [12].

Increasing evidence summarized in a systematic review by Shloim et al. shows that parental feeding practices were related to child weight status, mostly based on cross-sectional evidence [11]. In this context, restrictive feeding (i.e., parental restriction of their child’s access to food) was found to strengthen the child’s preference for the restricted food [13] and to be cross-sectionally related to higher standardized child body mass index (*z*BMI; [11]). Using food as reward (i.e., rewarding positive child behavior with food) was found to be positively associated with child *z*BMI in a cross-sectional study [14]. Another key parental feeding practice, pressure to eat (i.e., parental tendency to force their children to eat more), was found to create negative feelings to the pressured foods [13] and was negatively associated with child *z*BMI in cross-sectional studies [11]. For the association between parental monitoring of child’s food intake (i.e., parental oversight of their child’s eating) and child *z*BMI, cross-sectional studies provided ambiguous findings, mostly demonstrating lack of associations [15,16].

Concerning longitudinal findings, evidence is weaker compared to cross-sectional findings or inconsistent yet [11]. Specifically, restrictive feeding showed no prospective effects on child *z*BMI in two longitudinal studies in children aged 5 to 11 years [15,16], except in a study of *n* = 204 parents and their children indicating restrictive feeding assessed between the ages of 5 and 6 years at baseline to predict lower child *z*BMI at a 3-year follow-up [17]. Using food as reward in children aged 2 years at baseline was found to be prospectively associated with greater child *z*BMI gain 1 year later [18]. Further results on pressure to eat and child *z*BMI indicated bi-directionality, with a prospective relationship between greater pressure to eat and lower child *z*BMI in 1- to 2- and 5- to 7-year-old children [19,20] and a prospective association between lower child *z*BMI and higher use of pressure to eat between the ages of 7 and 11 [16]. The few existing evidence on the associations between parental monitoring and child *z*BMI was inconsistent, indicating a lack of association in 7- to 11-year-old children [16] or protective effects of monitoring in 5-year-old children on child *z*BMI at 7 years of age [15].

It is important to note that previous studies are predominantly cross-sectional in design [11], not allowing any conclusions whether parental feeding practices may be a cause or consequence of child *z*BMI. In addition, the few longitudinal studies carried out to date have concentrated mainly on predicting child *z*BMI by parental feeding practices, not considering the assumption that parental feeding practices can be a consequence of child weight status, too [21,22]. In this context, first studies analyzed the bi-directional effects of parental controlling feeding practices (i.e., restrictive feeding, pressure to eat, and monitoring) and objectively measured child *z*BMI in different European samples of *n* = 213 7- to 9-year-old children with a 3-year follow-up [16], *n* = 526 6- to 11-year-old children with a 10-month follow-up [21], *n* = 3708 4-year-old children with a 3-year follow-up [23], *n* = 4166 2-year-old children with two 2-year follow-ups [24], and *n* = 4689 4-year-old children with a 6-year follow-up [25]. Consistently, these studies suggested that controlling feeding practices are a consequence rather than a cause of child *z*BMI [16,21,23,24,25]. However, research that examines the entire childhood from early to middle childhood is still lacking.

Beyond the observation that parental feeding practices are associated with child *z*BMI [11] and that child weight status from the age of 5 years on is relatively stable and continuous into childhood, adolescence, and young adulthood [26,27,28,29], little is known about the stability (consistency of individual levels over time) and continuity (consistency of group levels over time) of parental feeding practices across childhood [30]. First evidence from relatively small samples (*n* = 31–183; [19,30,31,32,33]) suggested that there was high stability and continuity in parental controlling feeding practices assessed via the Child Feeding Questionnaire (CFQ; [12]), but these studies only examined narrow age ranges: 1–2 years [32], 3–4 years [31,33], 2–5 years [30], and 5–7 years [19]. A study in older children from a British population, however, indicated decreasing levels of parental controlling feeding practices in their children aged between 7 and 11 years [16], leaving the question whether the continuity of parental feeding practices changes from early to middle childhood.

Therefore, the aim of this study was to determine the stability and continuity of parental feeding practices and child *z*BMI, and their bi-directional associations in a large longitudinal population-based sample of 2- to 12-year-old German children. According to previous research mentioned above, it was hypothesized that (1) parental restrictive feeding, food as reward, pressure to eat, monitoring, and child *z*BMI are relatively stable and continuous during childhood, (2) restrictive feeding and child *z*BMI show a bi-directionally positive prospective association, (3) food as reward positively predicts child *z*BMI, while there is no effect of child *z*BMI on food as reward, (4) pressure to eat and child *z*BMI show a bi-directionally negative prospective association, and (5) child *z*BMI and parental monitoring are not related to each other over time.

## 2. Materials and Methods

### 2.1. Participants and Procedures

Study data were provided from the ‘Leipzig Research Center for Civilization Diseases (LIFE) Child’ study (‘LIFE Child Study’), which is a prospective, longitudinal population-based cohort study collecting data from fetal life until adulthood. In the ‘LIFE Child Study’, participants are recruited without further exclusion criteria at different time points, with recruitment age ranging between the 24th week of gestation and 16 years of child age, and reassessed at multiple follow-ups. The study was approved by the Ethics Committee of the University of Leipzig, Germany (reg. no. 264-10-19042010) and conducted in accordance with the Helsinki Declaration [34]. Advertisement in kindergartens, schools, health care practices (e.g., in hospitals, clinics, public health centers, and doctors’ surgeries), and by media (e.g., internet, radio, and television) was used for recruitment. At the time of the current study data of 2723 parent-child dyads were available. All parents were informed about the study in detail, data use, potential risks of participation, and the right to withdraw from the study without explanations or adverse consequences at any time, and signed informed written consent forms. All parents and children participated in comprehensive assessments (e.g., clinical examinations, questionnaires, and interviews) conducted by well-trained assessors (e.g., physicians, nurses, psychologists, and nutritional scientists) in a research center at the University Hospital of Leipzig at different time points. Monetary (≤ 20 Euro per child) and some further incentives (e.g., items with the study logo, feedback on results) served as a compensation for participation (for detailed information, see [35,36]). Eligible for the current study were *n* = 1512 child-parent dyads with one follow-up assessment at minimum assessed 10–14 months after baseline and valid data on the CFQ, standardized child BMI, child age, child sex, and baseline socio-economic status. Assessment was carried out at least twice in *n* = 328 children at age 2 to 3, *n* = 366 at age 3 to 4, *n* = 299 at age 4 to 5, *n* = 358 at age 5 to 6, *n* = 307 at age 6 to 7, *n* = 401 at age 7 to 8, *n* = 372 at age 8 to 9, *n* = 416 at age 9 to 10, *n* = 360 at age 10 to 11, and *n* = 363 at age 11 to 12.

### 2.2. Measures

#### 2.2.1. Child Feeding Questionnaire (CFQ)

The CFQ is a 31-item questionnaire used to assess three factors on parents’ attitudes and practices on child feeding and four factors on beliefs and concerns on child feeding and weight. For the current study, only the factors on parental feeding practices were used. The three factors on parental feeding practices from the original version by Birch et al. [12] contain restrictive feeding (eight items), pressure to eat (four items), and monitoring (three items). As recent psychometric analyses suggested the establishment of a fourth subscale, food as reward, consisting of two items of the original restriction subscale [37,38,39], restrictive feeding (six items) and food as reward (two items) were considered as separate parental feeding practices in the present study. All CFQ items are rated on a 5-point Likert scale recording agreement (1 = disagree to 5 = agree) or frequency (1 = never to 5 = always), with higher scores indicating greater controlling feeding practices and food-rewarding. Mean scores were used to quantify each of the four factors. The CFQ was gathered at all annual follow-up visits. The CFQ showed acceptable to excellent internal consistency of the subscales (Cronbach’s α = 0.71–0.91) in previous research using a subsample of this study’s sample (*n* = 982 mothers and their 2- to 13-year-old children; [37]).

#### 2.2.2. Child Characteristics

Children’s height and weight were measured by a stadiometer ‘Dr. Keller I’ (Längenmesstechnik Limbach GmbH, Limbach-Oberfrohna, Germany) and the calibrated scale ‘Seca 701’ (Seca Gmbh & Co. KG, Hamburg, Germany) at all visits. BMI (kg/m^2^) was transformed into BMI *z* scores (*z*BMI) according to sex- and age-specific references published by Kromeyer-Hauschild et al. [40]. Child sex and age were assessed by parent-report at baseline.

#### 2.2.3. Socio-Economic Status (SES)

The family socio-economic status (SES) was evaluated at all follow-up visits using the Winkler Index [41,42], ranging from 3 to 21. Based on income, education, and occupation families were classified as having low (3–8), medium (9–14), or high (15–21) SES.

### 2.3. Statistical Analyses

First, CFQ items were analyzed for missing values. Missing values were computed by the participant’s mean of the subscale in case of ≤ 25% missing values per subscale. Otherwise the participant was excluded from the analyses. Shapiro–Wilks tests of normal distribution were calculated for all variables. Based on outlier analyses, data which were three standard deviations under or above the mean of each variable were eliminated.

Stability of parental feeding practices and child *z*BMI was examined using Pearson product-moment or Spearman rank order correlations for normally or non-normally distributed data, respectively. Additionally, paired *t* tests and post-hoc effect size analyses using Cohen’s *d* were examined to analyze continuity of parental feeding practices and *z*BMI at the different visits and their follow-up. Using the software package G*Power (Version 3.1.9.2; [43]), a post-hoc power analysis showed that the estimated statistical power (1–β) for the paired *t* tests (*n* = 299–416, two-tailed α = 0.05) was 1.00 for detecting a medium (Cohen’s *d* = 0.5) or large effect (Cohen’s *d* = 0.8).

**Figure 1:**
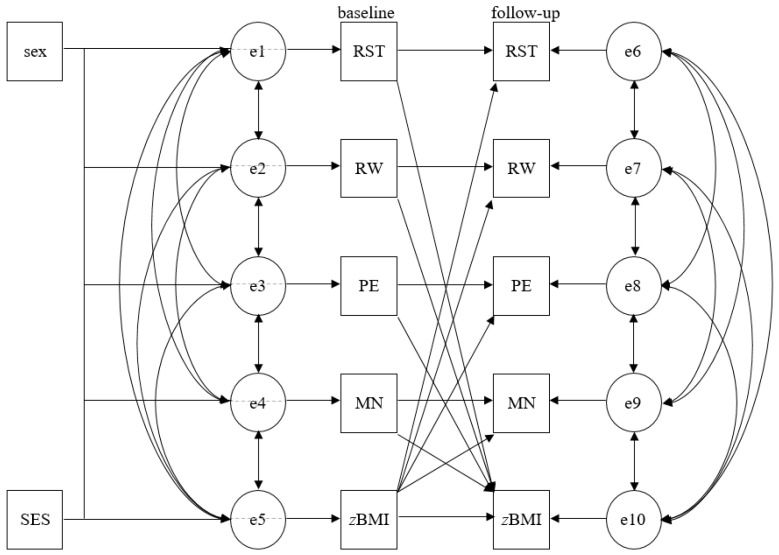
Cross-lagged panel analysis. sex—child sex; SES—socio-economic status; e—error term; RST—restrictive feeding; RW—food as reward; PE—pressure to eat; MN—monitoring; zBMI—standardized body mass index.

Cross-lagged panel design using structural equation modeling (SEM) was used to test the bi-directional associations between each parental feeding practice (i.e., restrictive feeding, food as reward, pressure to eat, and monitoring) and child *z*BMI. SEM was run as path analysis, including correlations to examine the cross-sectional associations within parental feeding practices and between each parental feeding practice and child *z*BMI, and linear regressions to analyze longitudinal associations within both variables, and the cross-lags between both variables from 2 to 11 years of age at baseline and the mean 1-year follow-up. In accordance with the literature, the models were adjusted for child sex and SES [23,24]. Figure 1 visualizes the cross-lagged panel analysis. Standardized βs were used to determine the relative strength of the examined associations. Model fit indices were calculated, with cut-offs in parentheses indicating good fit: χ^2^ (*p* > 0.05), root-mean-square error of approximation (RMSEA ≤ 0.05), comparative fit index (CFI ≥ 0.95), Tucker–Lewis index (TLI ≥ 0.95; [44]), and minimum discrepancy divided by its degrees of freedom (CMIN/df < 2).

A two-tailed *p* < 0.05 was set for statistical significance. Except for the SEM, which was calculated using Amos (Version 20.0; IBM SPSS, Inc., Chicago, IL, USA), statistical analyses were run using SPSS for Windows (Version 24.0; IBM SPSS, Inc., Chicago, IL, USA).

## 3. Results

### 3.1. Sample Characteristics

Within the total sample, all children (*n* = 1512) were assessed with multiple visits, ranging between two and eight visits (*M* = 3.74, *SD* = 1.40). Follow-up was conducted with a mean lag of 11.55 ± 0.79 months, ranging between 10 and 14 months. Age-specific sample characteristics are shown in Table 1.

### 3.2. Stability of Parental Feeding Practices and Standardized Child Body Mass Index

Table 2 shows the Pearson product-moment and Spearman rank order correlations between parental feeding practices and child *z*BMI assessed between 2 and 11 years and their mean 1-year follow-up. All variables were significantly correlated over time indicating moderate to high stability of parental feeding practices (*r* = 0.365–0.695, all *p*s < 0.01) and very high stability of child *z*BMI from baseline to follow-up (*r* = 0.818–0.951, all *p*s < 0.01).

### 3.3. Continuity of Parental Feeding Practices and Standardized Child Body Mass Index

Table 3 shows the results of the paired *t* tests used to examine continuity of parental feeding practices and child *z*BMI.

#### 3.3.1. Restrictive Feeding

Parental restrictive feeding significantly decreased between the ages of 3 to 4 (Cohen’s *d* = −0.124), but showed continuity otherwise.

#### 3.3.2. Food as Reward

Using food as reward statistically changed between the ages of 2 to 3, 5 to 6, 7 to 8, 8 to 9, and 11 to 12 (Cohen’s *d* = −0.183–0.175). While using food as reward rose between the ages of 2 to 3, it declined to all other follow-ups.

#### 3.3.3. Pressure to Eat

Parental pressure to eat statistically changed between the ages of 3 to 4, 9 to 10, and 11 to 12 (Cohen’s *d* = −0.180–0.170). While pressure to eat increased between the ages of 3 to 4, a decrease was observed for all other follow-ups in older age ranges.

#### 3.3.4. Monitoring

Parental monitoring statistically differed between the ages of 2 to 3, 4 to 5, 6 to 7, 7 to 8, 8 to 9, and 9 to 10 (Cohen’s *d* = −0.115–−0.151), consistently decreasing from baseline to follow-up.

#### 3.3.5. Standardized Child Body Mass Index

Child *z*BMI significantly changed between the ages of 3 to 4, 4 to 5, 7 to 8, 9 to 10, and 11 to 12 (Cohen’s *d* = −0.384–0.122). While *z*BMI declined in younger age ranges up to 8 years, it rose in older age ranges from 9 years up.

### 3.4. Bi-Directional Associations between Parental Feeding Practices and Standardized Child Body Mass Index

All cross-lagged panel designs used to analyze the bi-directional associations between each parental feeding practice and child *z*BMI presented a RMSEA close to 0.05 or ≤ 0.05, CFI ≥ 0.95, TLI close to 0.95 or ≥ 0.95, and CMIN/df close to 2 or < 2, supporting goodness of fit (Table 4).

#### 3.4.1. Restrictive Feeding

**Figure 2:**
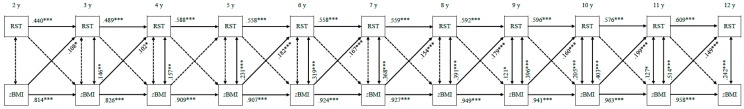
Bi-directional associations between restrictive feeding and standardized child body mass index. RST—restrictive feeding; *z*BMI—standardized body mass index. All associations are controlled for child sex and socio-economic status. Double-headed arrows display correlation coefficients between error terms; single-headed arrows display standardized regression coefficients; dotted arrows indicate non-significant associations. *** *p* < 0.001, ** *p* < 0.01, * *p* < 0.05.

Figure 2 shows the bi-directional associations between restrictive feeding and child *z*BMI. Except between the ages of 4 and 5, child *z*BMI consistently positively predicted restrictive feeding at follow-up (standardized β = 0.102–0.199, all *p*s < 0.05). Restrictive feeding at baseline did not significantly predict child *z*BMI at follow-up at any time (all *p*s ≥ 0.05).

#### 3.4.2. Food as Reward

**Figure 3:**
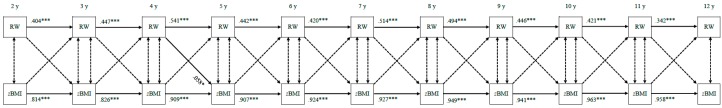
Bi-directional associations between food as reward and standardized child body mass index. RW—food as reward; *z*BMI—standardized body mass index. All associations are controlled for child sex and socio-economic status. Double-headed arrows display correlation coefficients between error terms; single-headed arrows display standardized regression coefficients; dotted arrows indicate non-significant associations. *** *p* < 0.001, * *p* < 0.05.

Figure 3 shows the bi-directional associations between using food as reward and child *z*BMI. Food as reward at 4 years predicted child *z*BMI at 5 years only (standardized β = 0.058, *p* = 0.025). Child *z*BMI at baseline did not significantly predict food as reward at follow-up at any time (all *p*s ≥ 0.05).

#### 3.4.3. Pressure to Eat

**Figure 4:**
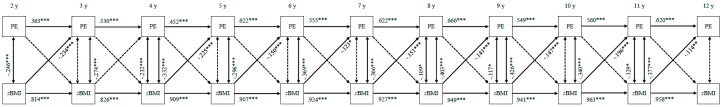
Bi-directional associations between pressure to eat and standardized child body mass index. PE—pressure to eat; *z*BMI—standardized body mass index. All associations are controlled for child sex and socio-economic status. Double-headed arrows display correlation coefficients between error terms; single-headed arrows display standardized regression coefficients; dotted arrows indicate non-significant associations. *** *p* < 0.001, ** *p* < 0.01, * *p* < 0.05.

Figure 4 displays the bi-directional associations between pressure to eat and child *z*BMI. Except between the ages of 3 and 4, child *z*BMI at baseline consistently negatively predicted pressure to eat at follow-up (standardized β = −0.114–−0.234, all *p*s < 0.01). Pressure to eat at baseline did not significantly predict child *z*BMI at follow-up at any time (all *p*s ≥ 0.05).

#### 3.4.4. Monitoring

**Figure 5:**
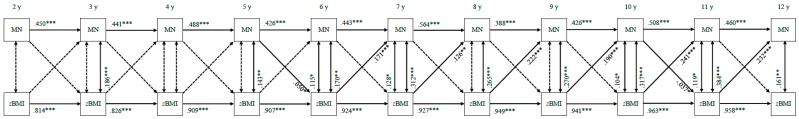
Bi-directional associations between monitoring and standardized child body mass index. MN—monitoring; *z*BMI—standardized body mass index. All associations are controlled for child sex and socio-economic status. Double-headed arrows display correlation coefficients between error terms; single-headed arrows display standardized regression coefficients; dotted arrows indicate non-significant associations. *** *p* < 0.001, ** *p* < 0.01, * *p* < 0.05.

Figure 5 depicts the bi-directional associations between monitoring and child *z*BMI. Child *z*BMI from the age of 6 onwards consistently positively predicted monitoring at follow-up (standardized β = 0.126–0.241, all *p*s < 0.05). Additionally, monitoring at 5 years significantly positively predicted child *z*BMI at 6 years (standardized β = 0.050, *p* = 0.033) and monitoring at 10 years significantly negatively predicted child *z*BMI at 11 years (standardized β = −0.037, *p* = 0.035).

## 4. Discussion

To our knowledge, this is the first prospective longitudinal study that examined the stability and continuity of parental feeding practices and standardized child BMI and their bi-directional associations in a large population-based sample across early and middle childhood, i.e., between the ages of 2 and 11 years, with a 1-year follow-up on average. As the main result, cross-lagged panel analysis showed that standardized child BMI predicted restrictive feeding, pressure to eat, and monitoring, supporting previous evidence of a child-effect model from studies with smaller age ranges [16,21,23,24,25]. There were no or only minor prospective associations found between food as reward and child *z*BMI. Consistently, parental feeding practices and child *z*BMI showed moderate to high stability across childhood. Continuity was high for restrictive feeding, and otherwise showed only minor temporal changes for the remaining feeding practices and standardized child BMI.

The result that parents used restrictive feeding practices in response to their child’s *z*BMI is consistent with a study in pre-school children [24]. Otherwise, the result contrasts two longitudinal studies that did not find an interaction between restrictive feeding and child *z*BMI in children between the ages of 2 to 5 and 7 to 11 which might be due to the relatively small sample sizes in these studies [16,30]. Inconsistency of results on the child *z*BMI–restriction relation might be explained by the interaction of restrictive feeding with other child characteristics (e.g., eating and activity style) as indicated in previous research showing higher restriction in 5- to 7-year-old children relatively high in hunger compared to their peers [15]. Future research is needed to examine if child *z*BMI solely or only in addition with other factors (e.g., child’s eating style or temperament) predicts parental restriction. Similar to previous studies [15,25,31], parental restriction did not predict child *z*BMI. Possibly, if parents restrict food at home, their children may seek out or binge eat food elsewhere (e.g., childcare, school, stores, social environment), counteracting the intended effect of parental restrictive feeding [13]. The lacking effect of parental restriction on child *z*BMI also suggests that other factors (e.g., activity style) rather than feeding practices may have a key influence on BMI development in children [1,10], highlighting the need for future research to provide enhanced understanding of risk factors influencing child BMI.

While higher standardized child BMI predicted more restrictive feeding, lower child *z*BMI predicted higher parental pressure to eat. This is in line with the hypothesis and previous prospective research showing strong evidence that pressure to eat is a reaction to lower child *z*BMI in 4- to 11-year-old children [16,23,24]. Similar to restrictive feeding, pressure to eat showed no prospective effect on child *z*BMI, contradicting previous longitudinal research with relatively small sample sizes in 1- to 2- and 5- to 7-year-old children [19,20]. Inconsistence in the results of the pressure to eat–child *z*BMI relation might be due to the different age ranges (i.e., 1- to 2- vs. 2- to 12-year-old children) or length of follow-up periods (i.e., 1 vs. 2 years). Pressure to eat possibly helps to prevent further decrease of child weight, however, the observational study design does not allow to know how the *z*BMI would have developed if the parents did not use pressure to eat. Experimental study designs are necessary to investigate the long-term influence of pressure to eat on child *z*BMI development.

The present study showed that higher standardized child BMI predicted higher levels of parental monitoring one year later in school-aged children between 6 and 11 years. In the literature, inconsistent or no effects were reported for the associations between monitoring and child *z*BMI [15,31], except in the study by Webber et al. [16] reporting that a higher child *z*BMI predicted higher monitoring in children aged 7 to 11 years. The child *z*BMI–monitoring relation found in this study is in line with research on covert control, a feeding practice moderately correlated with monitoring [45] which was cross-sectionally found to be associated with higher child weight status in *n* = 297 4- to 11-year-old British children [46]. Future research is needed to identify mediating factors on the child *z*BMI–monitoring relation. Notably, monitoring was found to predict child *z*BMI, but with weaker effects than child *z*BMI predicted monitoring. Specifically, monitoring predicted higher child *z*BMI between the ages of 5 and 6. This result is similar to previous cross-sectional research in *n* = 93 3- to 14-year-old children, which found a combined factor of monitoring and food-rewarding to be associated with higher child *z*BMI [14]. Additionally, monitoring predicted lower child *z*BMI in our study, but between the ages of 10 and 11 only. This could mean that the adaptation of parental feeding practices to a higher child *z*BMI resulted in a lower child *z*BMI one year later, which is in line with previous research in *n* = 174 Mexican American children aged 8 to 10 showing that more positive parental involvement in child food intake was associated with lower child *z*BMI [47]. Similarly, Jansen et al. [24] found a significant negative effect of monitoring at age 4 on child *z*BMI at age 6. However, when controlling for child age, child ethnicity, maternal education, and maternal BMI this effect was no longer significant. Future research is needed to clarify inconsistency of results on the monitoring–child *z*BMI association [11].

To our knowledge, this is the first study examining the bi-directional relationship between using food as reward and child *z*BMI. As a novel result, food as reward was found to predict child *z*BMI only between the ages of 4 and 5 years. Similarly, a study in 2-year-old children showed higher use of food as reward to predict higher weight status in children one year later [18]. The predictive effect of using food as reward on weight status in younger children only may reflect that this feeding practice is commonly used as a primary reinforcer in younger rather than older children to shape the child’s behavior [48]. Additionally, this age range is typical for “picky” eating behavior in children [49], potentially leading parents to use food as reward for eating non-preferred foods [50], which in turn might influence BMI development through an increased caloric intake. Typically, parents use palatable foods (e.g., sweets) as rewards, which are usually energy-dense and high in calories, to have a high rewarding value [51]. As frequent use of food as reward is known to enhance children’s responsiveness to food cues, refusal to try new foods, desire to eat, emotional overeating, loss of appetite for nutrient-dense foods [50], and dental caries [52], future research on the bi-directional association between food-rewarding and child BMI is needed to promote non-food rewarding strategies and thereby healthy child BMI and eating habits.

Stability of parental feeding practices from baseline to the 1-year follow-up was moderate to high confirming previous research in 1- to 7-year-old children with 1- to 3-year follow-ups even with comparable strength of correlation coefficients [19,30,31,32]. The result that stability of parental feeding practices was lowest in the 2- to 3-year-old children might be due to adaptations in parenting to child growth [49]. In addition, there are substantial variations in children’s food intake: for example, although children’s food spectrum strongly expands during this time of age, many children start “picky eating” [49]. Similarly to parental feeding practices, the finding that relative *z*BMI is stable in childhood is in line with evidence from an Australian sample in 5- to 10-year-old children with a 3-year follow-up [28]. Although parental feeding practices and child *z*BMI showed moderate to high stability, statistically significant increases or decreases in these variables were observed in different age ranges from baseline to follow-up (continuity), however, with small effects indicating only minor changes. As previous research has shown that relative weight increases overall across childhood and that child weight status shows high stability and continuity across childhood [29], the short length of the follow-up might not be enough time to detect changes in feeding practices and child *z*BMI. Thus, parental feeding practices and child *z*BMI generally seem to show fair continuity and were only subject to minor temporal changes due to the short follow-up and large sample size rather than clinically significant differences from baseline to follow-up [53].

A major strength of the present study is the large longitudinal population-based sample assessed prospectively at a wide age range using objective measurements of height and weight and an internationally established questionnaire with good psychometric properties [12] to gather data on parental feeding practices annually at multiple visits. Another strength is the separate analysis of restrictive feeding and food as reward as distinct feeding practices according to the current state of research on parental feeding practices (e.g., [37]). Although parent-child dyads were not assessed at every follow-up visit from the age of 2 years onwards, large subsamples were available for each age category. Nevertheless, it was not possible to analyze the development of parental feeding practices and child *z*BMI longitudinally over more than one year in the same sample, limiting the long-term perspective of our results. Although internationally established questionnaires were used to measure parental feeding practices, self-report measurement may be biased (e.g., via socially desirable response behavior). Objective measurement, for example, through video recordings of parental feeding practices, is, however, difficult to realize in a population-based cohort study. Additionally, it is possible that the high proportion of families with medium to high SES in our sample (see Table 1) is not entirely representative of the general German population, which is in line with previous research showing that a high SES is typically observed in population-based studies [54]. Further, the proportion of children with overweight and obesity in our sample (4.0%–17.9%) was lower than in the general German population (ca. 20%; [4]), probably caused in part by comparatively high SES in this study (see Table 1; [55]), which could also have influenced the results on feeding practices and child *z*BMI. In addition to SES and overweight, ethnicity might have influenced the study results, given cultural differences in parental feeding practices [56]. Notably, the study did not assess other potential confounders of child *z*BMI development and parental feeding practices, such as parental feeding practices from child birth to 2 years of age and non-parental child feeding elsewhere than at home (e.g., childcare, school, relatives; [11]).

Clinically, controlling feeding practices were found to not have the desired effect on decreasing or increasing child weight status and are known to have adverse consequences like disordered eating behaviors (e.g., loss of control eating or weight control behavior) in children in the short- and long-term [46,57]. Consequently, it is important to examine public health interventions that teach adequate feeding practices beneficial to healthy child weight status [58,59], in order to prevent negative child health development. Due to the high stability and continuity of parental feeding practices and child *z*BMI with slightly more fluctuations in early childhood, educational interventions should focus especially on parents with young children to promote healthy feeding practices from birth onwards and improve child nutrition across childhood. Additionally, longitudinal studies examining parental feeding practices in a more naturalistic manner (e.g., observational studies) are necessary to prevent bias resulting from self-report questionnaires (e.g., socially desirable response behavior). Further, experimental and interventional studies are warranted to examine cause and effect of parental feeding practices and child *z*BMI, and interventions that are best in promoting healthy child development, respectively. These further studies on the stability, continuity, and bi-directionality of parental feeding practices and child *z*BMI should be conducted with long-term follow-ups in order to depict the development over time. In addition, the use of heterogeneous populations (regarding SES, ethnicity, different genders of child caregivers) are recommended to enhance external validity of study results.

## 5. Conclusions

The current study showed moderate to high stability and individual patterns of continuity of parental feeding practices and standardized child body mass index. While our findings suggest that parents adapt their controlling feeding practices to child *z*BMI across all child ages, child *z*BMI was only positively affected by parental use of food as reward in pre-school children. Future research is warranted to examine early feeding practices and child *z*BMI from birth to 2 years onwards to validate whether a child-effect model can already be seen at this age range.

## Figures and Tables

**Table 1:** Mean and standard deviations of sample characteristics.

	Years of Age																			
	2 to 3 (*n* = 328)		3 to 4 (*n* = 366)		4 to 5 (*n* = 299)		5 to 6 (*n* = 358)		6 to 7 (*n* = 307)		7 to 8 (*n* = 401)		8 to 9 (*n* = 372)		9 to 10 (*n* = 416)		10 to 11 (*n* = 360)		11 to 12 (*n* = 363)	
**Children**																				
Sex % girls/boys	49.1/50.9		48.6/51.4		48.2/51.8		48.0/52.0		50.2/49.8		49.1/50.9		44.9/55.1		44.0/56.0		43.6/56.4		46.0/54.0	
Age	2.04 ± 0.16	3.05 ± 0.17	3.02 ± 0.21	4.03 ± 0.21	4.04 ± 0.26	5.04 ± 0.26	4.99 ± 0.27	5.99 ± 0.27	5.97 ± 0.28	6.98 ± 0.29	6.97 ± 0.29	7.97 ± 0.28	7.99 ± 0.28	9.00 ± 0.28	8.99 ± 0.27	10.00 ± 0.27	9.98 ± 0.28	10.99 ± 0.28	11.01 ± 0.29	12.02 ± 0.29
*z*BMI	0.24 ± 0.81	0.23 ± 0.74	0.20 ± 0.74	0.03 ± 0.73	−0.01 ± 0.77	−0.11 ± 0.79	−0.12 ± 0.82	−0.15 ± 0.87	−0.14 ± 0.84	−0.15 ± 0.85	−0.14 ± 0.90	−0.18 ± 0.91	−0.07 ± 0.97	−0.07 ± 1.01	0.01 ± 1.02	0.05 ± 1.02	0.08 ± 1.02	0.09 ± 1.05	0.18 ± 1.12	0.22 ± 1.14
*z*BMI %																				
underweight	1.2	0.6	0.3	0.3	0.7	0.3	0.8	1.1	0.7	0.3	1.0	1.0	0.3	1.1	1.2	1.7	0.8	2.2	1.7	1.1
normal weight	89.9	92.1	92.3	94.5	95.3	94.7	92.7	92.2	93.8	92.5	92.3	92.3	90.9	88.4	86.8	85.8	86.7	83.3	80.7	81.0
overweight	6.7	6.4	6.3	4.4	3.7	4.7	5.3	3.6	2.9	4.6	3.0	2.7	3.2	4.3	4.6	5.5	4.7	6.4	7.4	7.4
obesity	2.1	0.9	1.1	0.8	0.3	0.3	1.1	3.1	2.6	2.6	3.7	4.0	5.6	6.2	7.5	7.0	7.8	8.1	10.2	10.5
SES	13.79 ± 3.02		13.89 ± 3.13		14.24 ± 3.12		14.08 ± 3.13		13.96 ± 3.18		13.83 ± 3.28		13.62 ± 3.21		13.68 ± 3.39		13.42 ± 3.23		13.63 ± 3.32	
SES %																				
low	4.6		7.1		5.0		5.3		5.2		7.5		8.3		6.7		6.7		8.8	
medium	51.8		44.0		40.5		45.0		45.0		42.9		45.2		48.3		51.9		47.4	
high	43.6		48.9		54.5		49.7		49.8		49.6		46.5		45.0		41.4		43.8	
**CFQ**																				
RST	2.67 ± 0.84	2.70 ± 0.79	2.70 ± 0.82	2.60 ± 0.83	2.54 ± 0.80	2.49 ± 0.80	2.51 ± 0.83	2.42 ± 0.82	2.35 ± 0.82	2.33 ± 0.84	2.31 ± 0.85	2.23 ± 0.85	2.32 ± 0.85	2.31 ± 0.84	2.35 ± 0.89	2.29 ± 0.93	2.32 ± 0.90	2.29 ± 0.89	2.31 ± 0.88	2.28 ± 0.87
RW	2.05 ± 1.06	2.25 ± 1.08	2.17 ± 1.05	2.10 ± 1.10	2.07 ± 1.06	1.99 ± 1.04	2.05 ± 1.07	1.84 ± 0.97	1.78 ± 0.91	1.78 ± 0.92	1.76 ± 0.89	1.66 ± 0.87	1.71 ± 0.92	1.60 ± 0.86	1.64 ± 0.87	1.56 ± 0.86	1.63 ± 0.85	1.54 ± 0.79	1.51 ± 0.76	1.37 ± 0.65
PE	1.83 ± 0.82	1.85 ± 0.85	1.83 ± 0.82	1.96 ± 0.88	1.99 ± 0.87	1.92 ± 0.90	2.00 ± 0.93	1.99 ± 0.96	1.95 ± 0.95	1.95 ± 0.91	1.97 ± 0.95	1.93 ± 0.94	1.94 ± 0.96	1.90 ± 0.97	1.87 ± 0.93	1.76 ± 0.88	1.75 ± 0.85	1.75 ± 0.88	1.74 ± 0.86	1.60 ± 0.81
MN	4.01 ± 1.00	3.89 ± 0.97	3.92 ± 0.97	3.86 ± 1.01	3.78 ± 1.02	3.66 ± 0.98	3.67 ± 1.01	3.63 ± 1.02	3.72 ± 1.03	3.56 ± 1.06	3.61 ± 1.06	3.48 ± 1.05	3.59 ± 1.03	3.43 ± 1.04	3.52 ± 1.02	3.37 ± 1.10	3.44 ± 1.08	3.35 ± 1.05	3.43 ± 1.02	3.34 ± 1.01

Note: Except for sex, *z*BMI %, and SES %, depicting percent, all values represent means ± standard deviations for baseline assessment and annual follow-up within each age group. *z*BMI—standardized body mass index; SES—socio-economic status; CFQ—Child Feeding Questionnaire rated on a 5-point Likert scale (1 to 5), with higher scores indicating higher controlling feeding practices and food-rewarding; RST—restrictive feeding; RW—food as reward; PE—pressure to eat; MN—monitoring.

**Table 2:** Stability of parental feeding practices and standardized child body mass index.

	Years of Age									
Variable	2 to 3	3 to 4	4 to 5	5 to 6	6 to 7	7 to 8	8 to 9	9 to 10	10 to 11	11 to 12
CFQ										
RST	0.431	0.520	0.593	0.613	0.638	0.629	0.655	0.684	0.672	0.695
RW	0.416	0.476	0.552	0.429	0.445	0.557	0.482	0.424	0.403	0.365
PE	0.391	0.563	0.490	0.637	0.570	0.656	0.683	0.559	0.569	0.592
MN	0.458	0.491	0.481	0.417	0.448	0.576	0.473	0.483	0.584	0.542
*z*BMI	0.818	0.821	0.914	0.890	0.916	0.912	0.934	0.948	0.940	0.951

Note: All values represent *r*-correlation coefficients. All correlations shown were significant at a level of *p* < 0.01. CFQ—Child Feeding Questionnaire; RST—restrictive feeding; RW—food as reward; PE—pressure to eat; MN—monitoring; *z*BMI—standardized body mass index.

**Table 3:** Continuity of parental feeding practices and standardized child body mass index.

	Years of Age									
Variable	2 to 3	3 to 4	4 to 5	5 to 6	6 to 7	7 to 8	8 to 9	9 to 10	10 to 11	11 to 12
CFQ										
RST	0.68 (327)	2.12 (365) *	0.85 (298)	1.03 (357)	0.53 (306)	1.76 (400)	−0.56 (371)	1.24 (415)	0.28 (359)	−0.15 (362)
RW	−3.13 (327) **	1.32 (365)	1.46 (298)	3.71 (357) ***	0.15 (306)	2.34 (400) *	2.32 (371) *	1.76 (415)	1.90 (359)	3.27 (362) **
PE	−0.34 (327)	−3.18 (365) **	1.34 (298)	0.09 (357)	−0.15 (306)	1.11 (400)	1.25 (371)	2.84 (415) **	0.00 (359)	3.72 (362) ***
MN	2.14 (327) *	1.12 (365)	2.10 (298) *	0.70 (357)	2.58 (306) **	2.83 (400) **	2.78 (371) **	2.87 (415) **	1.78 (359)	1.74 (362)
*z*BMI	0.33 (327)	7.32 (365) ***	5.61 (298) ***	1.43 (357)	0.74 (306)	2.29 (400) *	0.07 (371)	−2.66 (415) **	−0.95 (359)	−2.44 (362) *

Note: All values represent *t*(df). CFQ—Child Feeding Questionnaire; RST—restrictive feeding; RW—food as reward; PE—pressure to eat; MN—monitoring; *z*BMI—standardized body mass index. *** *p* < 0.001, ** *p* < 0.01, * *p* < 0.05.

**Table 4:** Model fit indices of the cross-lagged panel analyses to examine bi-directionality of parental feeding practices and standardized child body mass index.

	*n*	χ^2^(df)	RMSEA (*p* Close)	RMSEA 95% CI		CFI	TLI	CMIN/df
Years of Age				Lower	Upper			
2 to 3	328	24.272 (23)	0.013 (0.962)	0.000	0.048	0.998	0.996	1.055
3 to 4	366	48.002 (23) **	0.055 (0.337)	0.033	0.076	0.978	0.936	2.087
4 to 5	299	25.104 (23)	0.018 (0.936)	0.000	0.052	0.998	0.995	1.091
5 to 6	358	41.822 (23) *	0.048 (0.529)	0.023	0.071	0.987	0.963	1.818
6 to 7	307	44.900 (23) **	0.056 (0.321)	0.031	0.080	0.984	0.953	1.952
7 to 8	401	40.227 (23) *	0.043 (0.668)	0.019	0.065	0.992	0.976	1.749
8 to 9	372	24.075 (23)	0.011 (0.978)	0.000	0.045	0.999	0.999	1.047
9 to 10	416	44.411 (23) **	0.047 (0.553)	0.026	0.068	0.990	0.972	1.931
10 to 11	360	37.133 (23) *	0.041 (0.699)	0.013	0.065	0.993	0.979	1.614
11 to 12	363	43.443 (23) **	0.050 (0.482)	0.026	0.072	0.990	0.972	1.889

Note: *n*—number of analyzed parent-child dyads. χ^2^(df)—chi-square test for goodness of fit (degrees of freedom); RMSEA—root-mean-square error of approximation; CI—confidence interval; CFI—comparative fit index; TLI—Tucker–Lewis index; CMIN/df—minimum discrepancy divided by its degrees of freedom. ** *p* < 0.01; * *p* < 0.05. coefficients between error terms; single-headed arrows display standardized regression coefficients; dotted arrows indicate non-significant associations. ** *p* < 0.01, * *p* < 0.05.

## Abbreviations

BMI	body mass index
CFQ	Child Feeding Questionnaire
LIFE	Leipzig Research Center for Civilization Diseases
MN	monitoring
PE	pressure to eat
RST	restrictive feeding
RW	food as reward
SEM	structural equation modeling
SES	socio-economic status
*z*BMI	standardized body mass index
